# Interaction Between Macrophage Extracellular Traps and Colon Cancer Cells Promotes Colon Cancer Invasion and Correlates With Unfavorable Prognosis

**DOI:** 10.3389/fimmu.2021.779325

**Published:** 2021-12-01

**Authors:** Tianli Chen, Yue Wang, Zhaodi Nan, Jie Wu, Ailu Li, Tingguo Zhang, Xun Qu, Chen Li

**Affiliations:** ^1^ Department of General Surgery, Qilu Hospital, Cheeloo College of Medicine, Shandong University, Jinan, China; ^2^ Department of Colorectal Surgery, National Cancer Center/National Clinical Research Center for Cancer/Cancer Hospital, Chinese Academy of Medical Sciences and Peking Union Medical College, Beijing, China; ^3^ Institute of Basic Medical Sciences, Qilu Hospital, Cheeloo College of Medicine, Shandong University, Jinan, China; ^4^ Department of Urology, National Cancer Center/National Clinical Research Center for Cancer/Cancer Hospital, Chinese Academy of Medical Sciences and Peking Union Medical College, Beijing, China; ^5^ Department of Obstetrics and Gynecology, Quanzhou First Hospital Affiliated to Fujian Medical University, Quanzhou, China; ^6^ Department of Pathology, Qilu Hospital, Cheeloo College of Medicine, Shandong University, Jinan, China; ^7^ Department of Ultrasound, Qilu Hospital, Cheeloo College of Medicine, Shandong University, Jinan, China

**Keywords:** colon cancer, macrophage extracellular traps (METs), prognosis, PAD2, PAD2-IN-1

## Abstract

**Background:**

Macrophage extracellular traps (METs) and tumor-infiltrating macrophages contribute to the progression of several diseases. But the role of METs and tumor-infiltrating macrophages in colon cancer (CC) has not been illuminated. In this study, we aimed to clarify the prognostic value of METs for CC patients and to explore the interaction between CC cells and METs *in vitro and in vivo*.

**Methods:**

A training cohort consisting of 116 patients and a validation cohort of 94 patients were enrolled in this study. Immunofluorescence (IF) staining was conducted to determine METs formation in CC patients. Cox regression was used to perform prognostic analysis and screen out the best prognostic model. A nomogram was established to predict 5-year overall survival (OS). The correlation between METs with clinicopathological features and inflammatory markers was analyzed. The formation of METs *in vitro* was detected by SYTOX^®^ green and IF staining, and the effect of METs on CC cells was detected by transwell assays. PAD2-IN-1, a selective inhibitor of peptidylarginine deiminase 2 (PAD2), was introduced to destroy the crosstalk between CC cells and METs *in vitro and in vivo*.

**Results:**

METs levels were higher in CC tissues and were an independent prognostic factor for CC patients. The prognostic model consisting of age, tumors local invasion, lymph node metastasis and METs were confirmed to be consistent and accurate for predicting the 5-year OS of CC patients. Besides, METs were correlated with distant metastasis and inflammation. Through *in vitro* experiments, we confirmed that there was a positive feedback loop between CC cells and METs, in that METs promoted the invasion of CC cells and CC cells enhanced the production of METs, in turn. This interaction could be blocked by PAD2-IN-1 inhibitors. More importantly, animal experiments revealed that PAD2-IN-1 inhibited METs formation and CC liver metastasis *in vivo*.

**Conclusions:**

METs were the potential biomarker of CC patient prognosis. PAD2-IN-1 inhibited the crosstalk between CC cells and METs *in vitro and in vivo*, which should be emphasized in CC therapy.

## Introduction

Colorectal cancer (CRC) is one of the most common malignancies, which can be further divided into colon cancer (CC) and rectal cancer (RC). According to the global cancer statistics in 2020, the incidence of CRC ranks third, and the mortality rate ranks second among all cancers (1, 2). With continuous improvement of early screening and treatment strategies for CRC, the prognosis of patients with orthotopic CRC has significantly improved. However, the prognosis of patients with metastatic CRC (mCRC) who have received postoperative adjuvant chemoradiotherapy and radiofrequency therapy is still poor, with a 5-year overall survival (OS) rate less than 15%, which is the main challenge for CRC treatment at present (2).

With the development of molecular biology technology, new therapeutic methods such as molecular targeted therapy have been proposed for CRC patients. But improvement of the survival rate and therapeutic effects are still unsatisfactory for patients with advanced CRC, especially those with distant metastasis (3). One of the primary reasons is that the drugs used to treat CRC are mainly targeted at tumor cells. However, tumors are not only composed of a group of homologous malignant cells, but can be considered a special ‘organ’, which is composed of tumor cells and other heterogeneous cells (4). Non-malignant cells such as stromal cells, immune cells, and other cells infiltrate the tumor, and the dynamic interaction network among the different cell sub-classes regulates the biological behavior of tumor cells and ultimately determines tumor progression, the prognosis of tumor patients, and the response to the onco-therapy. Immune cells constitute a very important non-malignant cell population in tumors (5, 6). CRC is an immune sensitive tumor, with numerous immune cells infiltrating the tumor microenvironment (7). Therefore, the targeted treatment of immune cells in CRC should be emphasized and studies on more effective predictors related to immune cells are necessary, in order to greatly improve the survival rate of CRC patients, especially those with distant metastasis.

Macrophages exist in and around many solid tumors and together with other important immune cells, such as NK cells, T cells, and neutrophils, constitute up to 80% of tumor infiltrating immune cells. Macrophages differentiate from peripheral blood monocytes when recruited to tissues and are the largest proportion of myeloid immune cells in tumor tissues. The main function of macrophages is to recognize and phagocytize antigens and present them to T cells, as well as regulate the activity of the acquired immune response. Macrophages are thus considered the bridge between innate immunity and acquired immune response (8, 9). Moreover, macrophages produce different tumor-promoting factors, such as reactive oxygen species, growth factors, and angiogenic factors, which promote tumor invasion and metastasis (10).

Plasticity is a widely accepted feature of bone marrow cells, especially the monocyte-macrophage system (11). With more thorough research, scientists have found that macrophages are involved in the pathological process of many diseases in the form of extracellular traps (ETs), also referred to as macrophage extracellular traps (METs). This discovery has gone beyond the traditional theory that macrophages can polarize into M1 (classical macrophages) and M2 (alternative activated macrophages) phenotypes under different environmental stimuli (12, 13). The formation of ETs is a type of cell death that is different from apoptosis or necrosis. It is characterized by the release of depolymerized chromatin from immune cells and the formation of a reticular structure after being activated by some stimulating factors (13). Besides the DNA skeleton, ETs also contain histone, granule protein, and other proteins (14). At present, research on ETs mainly focuses on neutrophil extracellular traps (NETs), which promote the progress of a variety of tumors and correlate with poor patient prognosis (15–19). METs have been reported to be involved in the response process of bacteria and their toxins, the lipid-rich lesions in atherosclerosis, and acute kidney injury (20–23). However, the research about METs in the field of cancer is limited to nonfunctional pancreatic neuroendocrine tumors, and there is little relevant research in CRC (24).

About 70% of CC patients present with macrophage infiltration, but its role in the progression of CC is still controversial. Some reports suggest that tumor infiltrating macrophages promote tumor progression and metastasis. Other reports show that intratumoral macrophages have the advantage of prolonged survival (7, 25). In CC, the role of METs, a special cell death form of macrophages, in the progression and metastasis of CC needs to be further elucidated. In this study, we first described the correlation between METs with the prognosis of patients with CC, and confirmed that presence of METs was an independent risk factor for the prognosis of CC. Additionally, we elucidated the interaction between METs and CC cells *in vitro*, showing that METs promoted the invasion of CC cells and CC cells also promoted the formation of METs. More importantly, we found that PAD2-IN-1, a selective inhibitor of PAD2, destroyed the interaction between METs and CC cells *in vitro* and inhibited METs formation and colon cancer liver metastasis *in vivo*. Our study provided a new target for the treatment of CC patients, especially those with distant metastasis, and also identified a new idea for CC immunotherapy.

## Methods and Materials

### Patients and Specimens

The training cohort consisting of 116 patients was selected following the criteria: (i) patients who underwent radical resection with a clear surgical margin; (ii) patients with available formalin-fixed tumor tissues, follow-up information and complete medical records; (iii) patients with a postsurgical survival time of more than 1 month; and (iv) patients with no history of other malignancies. The validation cohort of 94 patients was purchased from Shanghai Outdo Biotech Company. The tumors were classified and staged according to the 8th AJCC/UICC TNM classification system. Informed consent was obtained from all patients. All experiments were approved and supervised by the Ethics Committee of Qilu Hospital of Shandong University.

The clinical features of all the patients are described in **Supplemental Table 1**.

### Immunofluorescence Staining of Paraffin-Embedded Tissues

To visualize METs in patients’ tissue samples, paraffin-embedded tissue sections and TMA were subjected to immunofluorescence (IF) staining. METs were specifically detected by mouse anti-CD68 (ab955, 1:200, Abcam) and rabbit anti-Citrullinated histone H3 (H3Cit, ab5103, 1:200, Abcam) antibodies. Tris-EDTA was used for antigen retrieval and 5% bovine serum albumin (BSA) was used for nonspecific antigen blocking at room temperature. The sections were incubated with anti-CD68 and anti-H3Cit primary antibodies at 4°C overnight. After that, the sections were washed with PBS and incubated with CY3-conjugated goat anti-mouse (1:200, Servicebio) and Alexa Fluor^®^ 488-conjugated goat anti-rabbit (1:200, Servicebio) secondary antibodies at room temperature for 2 hours. Finally, the sections were mounted in Anti-fade Mounting Medium with DAPI (Beyotime Biotechnology).

IF assessments were performed by two independent experienced pathologists. METs were defined as DNA-, CD68- and H3Cit-positive MET fibers exceeding 20 μm in length (23), and macrophages were defined as co-localized CD68 and DNA without H3Cit. 5 random fields under HPF were selected over the whole section for the quantification of macrophages or METs in tumor and para-tumor tissues. Extracellular structures exceeding 20 μm in length with DNA-, CD68- and H3Cit-positive staining were calculated, manually. The mean value was regarded as the final score of macrophages infiltration or METs formation in the tumor of each patient, as described (24).

### Cell Lines and Agents

Human colon cancer cell lines HCT116 and SW480 were purchased from the Cell Bank of Chinese Academy of Sciences. HCT116 and SW480 were cultured in Dulbecco’s modified Eagle’s medium (DMEM, Hyclone) with 10% fetal bovine serum (FBS, Gibco Life Technologies), 100 U/ml penicillin, and 100 μg/ml streptomycin at 37°C under 95% air and 5% CO_2_. All cell lines were authenticated using short tandem repeat (STR) analysis, and the databases of the Chinese Academy of Sciences and American Type Culture Collection were used as references.

The PAD2 inhibitor PAD2-IN-1 was purchased from MedChemExpress (MCE, HY-136557). DNase I was purchased from ThermoFisher Scientific.

### Monocytes Isolation and Macrophages Culture

Monocytes isolation and macrophages culture were performed as previously described (26). Briefly, human monocytes were isolated from the peripheral blood of healthy donors using Ficoll density gradient centrifugation. Under sterile conditions, monocytes were resuspended in serum-free RPMI-1640 medium and seeded into 12-well culture plate in the density of 5 × 10^5^/ml. After 2 hours incubation at 37°C under 95% air and 5% CO_2_, monocytes will adhere to the culture plate. The cell medium was then replaced with complete RPMI-1640 culture medium containing 10% (v/v) pooled human serum and 20 mM L-glutamine and cultured for 8 days.

### *In Vitro* METs Formation Assay

To prepare the METs inducing medium, phorbol 12-myristate 13-acetate (PMA) was added to complete RPMI-1640 medium and the final concentration was adjusted to 5 μM. Under sterile conditions, the medium was removed from the culture plate wells and the wells were washed with PBS three times. Approximately 1 mL of METs inducing medium was added to each well containing macrophages. After 12 hours of incubation, METs were measured using SYTOX^®^ Green (S7020, Thermo Fisher Scientific) and IF staining.

For IF staining, cells were fixed with 4% paraformaldehyde at 4°C for 15 minutes and the nonspecific antigen was blocked with 5% goat serum at 37°C for 1 hours. Primary mouse anti-CD68 (ab955, 1:200, Abcam) and rabbit anti-H3Cit (ab5103, 1:200, Abcam) antibodies were applied and incubated at 4°C overnight. CY3-conjugated goat anti-mouse (1:200, Servicebio) and Alexa Fluor^®^ 488-conjugated goat anti-rabbit (1:200, Servicebio) secondary antibodies were used for 2 hours at room temperature. The percentage of the field of view positive for the green signal (H3Cit) was regarded as the level of METs formation and was measured using Image J (NIH).

SYTOX^®^ Green was also introduced to detect the extracellular DNA chains. SYTOX^®^ Green was diluted to 2 μM with RPMI-1640 and was added into each well. After 30 minutes of incubation at 37°C, the green signal was determined using a fluorescence microscope (Olympus IX70).

### Conditioned Medium Preparation

The preparation of conditioned medium (CM) was performed as described (27). Briefly, HCT-116 cells were cultured in DMEM medium with 10% FBS until the cell density became 40%-50%. Then replace the complete medium with serum-free medium and incubate the cells for 48 hours. The supernatant of the medium was collected and centrifuged for 1000g to discard the pellets. The supernatant was ten times concentrated with Amicon Ultra 15ml filters at 4000 g.

To determine whether colon cancer cells could induce METs, macrophages were stimulated with the CM from HCT116 cells for 12 hours at 37°C. METs were then visualized and assessment through SYTOX^®^ Green and IF staining as above described.

### Transwell Assays

Transwell assays were performed using 24 plates with transwell chambers (8 µm pore diameter, Corning). After METs were induced in the lower chamber, 5 × 10^4^ HCT-116 cells or 2 × 10^4^ SW480 cells were seeded to the upper chambers with matrigel coated (diluted at 1:6 with DMEM; Corning). After 36 hours, the cells attached to the bottom of the chamber were fixed with methanol for 20 minutes and then stained with 0.5% crystal violet (Beyotime) for 30 minutes at room temperature. Images of 5 random visual fields of microscopy at ×200 magnification were exported to Image J (NIH) for cell counting.

### Western Blotting

Western blotting assay was performed as described (27, 28). Briefly, total protein was extracted using RIPA lysis buffer (Beyotime) with 1% PMSF (Beyotime) and 1% phosphatase inhibitor (Solarbio). Protein concentration was determined using a bicinchoninic acid (BCA) Assay Kit (Beyotime). Protein extracts were resolved through 10% SDS-PAGE after denaturation, transferred to PVDF membranes (0.22µm, Millipore) and probed with rabbit anti-H3Cit (ab5103, 1:1000, Abcam) and rabbit anti-H3 (ab1791, 1:5000, Abcam) primary antibodies at 4°C overnight. The membranes were then incubated in the second antibody for 1 hour at room temperature. An enhanced chemiluminescence (Millipore) were used for HRP detection. Quantitative analysis of Western blotting bands was performed using Image J (NIH).

### *In Vivo* Experiments

Rat anti–mouse Ly6G antibody (BP0075-1, BioXcell) was used for neutrophil depletion (100 μg/mice, i.p.) as described (23). 5×10^5^ MC-38 cells were injected into the caudal vein of each BALB/c mouse, in the presence or absence of PAD2-IN-1 (20 mg/kg, i.p.). The presence of METs was detected by IF staining, as above described. The weights of livers were measured to assess the actual tumor burden. The number of nodules on the livers was counted and confirmed by HE staining. All animal experiments were approved by the Medical Ethics Committee of Shandong University.

### Statistical Methods

SPSS 17.0 and GraphPad Prism 5.0 software were used for statistical analysis and chart generation. The survival curves were plotted using the Kaplan-Meier method, and the log-rank test was conducted to determine the statistical significance. The Harrell’s concordance index (C-index) was calculated using “survival” of R package by RStudio software. The independent prognostic factors were analyzed in Cox proportional hazards regression model. The nomogram was established to predict 5-year OS, and the calibration plot was used to display the accuracy and predictive value of the prognostic models. Student’s t test was used for the comparison of two independent groups. Paired t test was used for the comparison of two paired groups. p values < 0.05 was considered statistically significant.

## Results

### Patient Characteristics

In total, 116 patients with CC were included in the training cohort and 94 patients with CC were included in the validation cohort. Variables of clinicopathological characteristics of patients are shown in **Supplemental Table 1**.

In the training cohort, the median age of 116 patients was 64 years old (ranging from 30 to 91), and 33.6% of these patients were female. A total of 102 patients (87.9%) had T3/T4 tumors, while 45 patients (38.8%) had lymph node metastasis (LNM) and 11 patients (9.5%) had distant metastasis, and all of them were in stage III/IV.

In the validation cohort, the median age of 94 patients was 65 years old (ranging from 27 to 90), and 48.9% of these patients were female. In total, 83 patients (88.3%) had T3/T4 tumor, 34 patients (36.2%) had LNM, and 5 patients (5.32%) had distant metastasis, and all of them were in stage III/IV.

### Prognostic Value of METs for CC Patients

**Figure 1:**
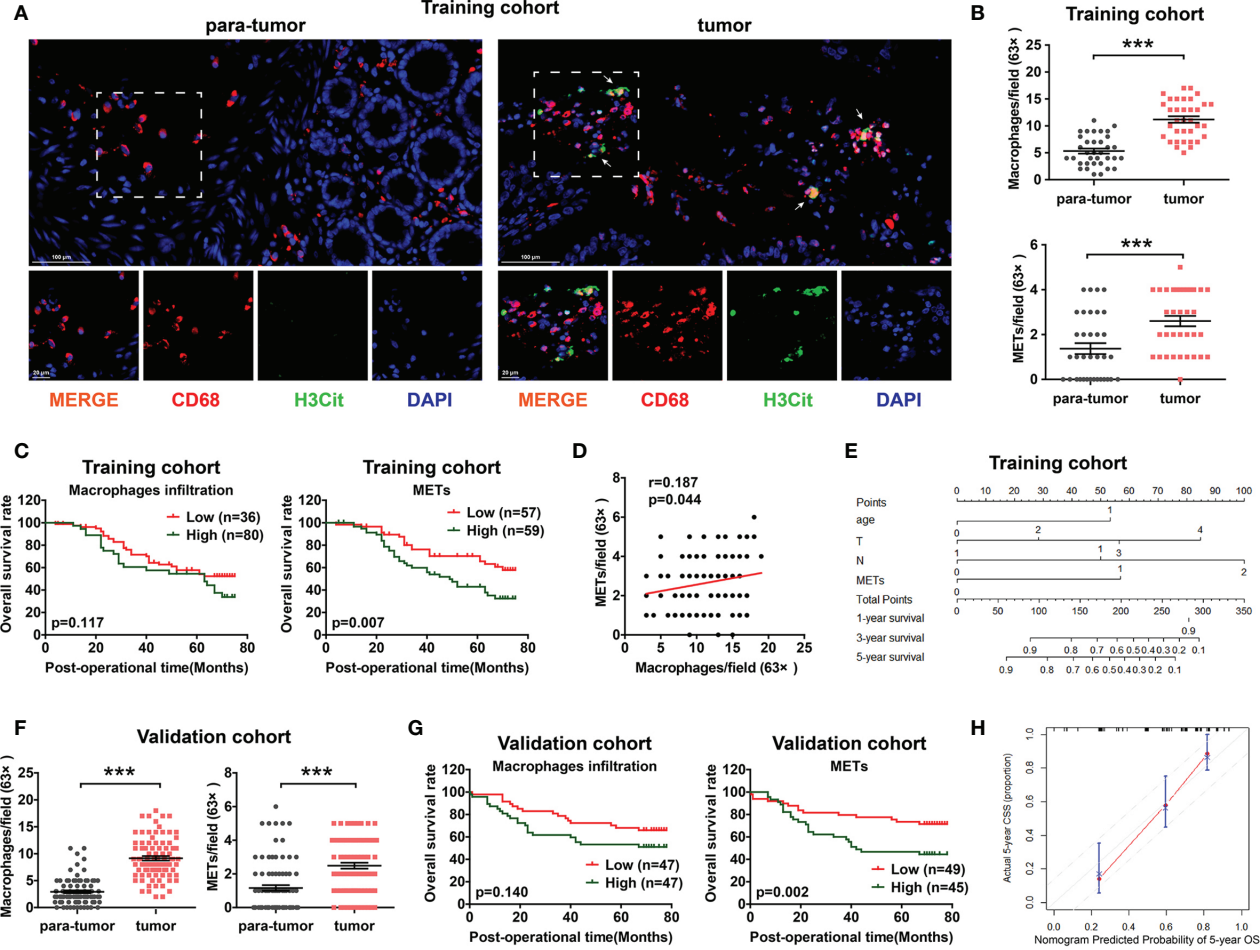
The prognostic value of METs for CC patients in training cohort and validation cohort. **(A)** Representative images of immunofluorescence staining of METs in CC patients. Arrows indicated the presence of METs. **(B)** In the training cohort, macrophage infiltration (upper panel) and METs (lower panel) were higher expressed in CC tissues than in para-tumor tissues. **(C)** High METs levels were correlated with poor prognosis of CC patients in training cohort (right panel), while macrophage infiltration had no prognostic value (left panel). **(D)** No significant correlation was observed between macrophage infiltration and METs formation in training cohort. **(E)** A nomogram for 5-year OS predictive probability. **(F)** In the validation cohort, macrophage infiltration (left panel) and METs (right panel) were higher expressed in CC tissues than those in para-tumor tissues. **(G)** The level of METs rather than macrophage infiltration correlated with poor prognosis of CC patients in validation cohort. **(H)** Calibration plot for 5-year overall survival. *** represents p<0.001. In **(B, F)**, data were calculated by Paired t test. In **(C, G)**, data were calculated by long-rank test. In **(D)**, data were calculated by Pearson correlation test.

To determine the levels of macrophage infiltration and METs formation in CC, we performed immunofluorescence (IF) staining on a total of 116 patients as the training cohort (**Figure 1A**). Both macrophage infiltration and METs presented with significantly higher levels in CC tissues than those in para-tumor tissues (**Figure 1B** and **Supplemental Figure 1A**). Univariate Cox regression analysis was performed to analyze the prognostic value of macrophage infiltration, METs, and other clinicopathological factors. Results showed that in CC, METs (p=0.007) exhibited a better prognostic value than macrophage infiltration (p=0.117) (**Figure 1C**). There was no correlation between macrophage infiltration and METs formation (**Figure 1D** and **Supplemental Figures 1B, C**). The tumor local invasion (T stage, p=0.020), positive lymph node metastasis (N stage, p<0.001), distant metastasis (M stage, p<0.001), and advanced TNM stage (p<0.001) were also strongly correlated with the poor prognosis (**Table 1**).

**Table 1:** Univariate and multivariate Cox regression analysis of prognostic factors for overall survival in CC.

Clinicopathological characteristics	Univariate Cox			Multivariate Cox		
	HR	95% CI	p value	HR	95% CI	p value
Age (< 60/≥ 60), years old	1.67	0.93-3.00	0.084	1.84	0.98-3.49	0.059
Gender (male/female)	0.72	0.42-1.24	0.241			
Tumor size (< 5/≥ 5), cm	1.30	0.76-2.20	0.340			
T stage (1/2/3/4)	1.63	1.08-2.45	0.020*	1.53	0.92-2.17	0.057
N stage (0/1/2)	2.14	1.57-2.93	<0.001*	1.88	1.36-2.82	<0.001*
M stage (0/1)	4.21	2.14-8.30	<0.001*	1.91	0.91-4.67	0.124
TNM stage (1/2/3/4)	2.69	1.92-3.79	<0.001*			
Macrophages infiltration (< 14/≥ 14)	1.54	0.90-2.64	0.117	1.45	0.87-2.67	0.195
METs (< 4/≥ 4)	2.11	1.22-3.64	0.007*	2.18	1.43-4.61	0.007*

HR, hazard ratio; 95% CI, 95% confidence interval.

Calculated by Cox-regression Hazard model. * means p<0.05.

Multivariate Cox regression analysis was further performed to identify the independent prognostic factors. All factors with p value ≤0.20 in univariate analysis were included in multivariate analysis, except TNM stage because of its natural interaction with T, N, or M stage. We found that presence of METs was an independent prognostic factor in CC (p=0.007). Patients with higher METs had 2.18 fold higher risk of cancer-caused death than those with lower METs (**Table 1**). In addition, we chose prognostic factors with p value ≤0.10 in multivariate analysis to establish a prognostic model including age, T stage, N stage, and METs. Concordance index (C-index) was used in our study to assess the consistency and accuracy of predictive models. This model showed the highest C-index (0.730), surpassing TNM stage and any single clinicopathological factors (**Table 2**). Thus, METs combined with age, T stage, and N stage were selected for the establishment of a nomogram to predict the 5-year overall survival rate (**Figure 1E**).

**Table 2:** Comparisons of prognostic models for overall survival in CC.

Prognostic models	C-index	
	Training cohort	Validation cohort
Age (< 60/≥ 60), years old	0.559 (se = 0.033)	0.503 (se = 0.038)
T stage (1/2/3/4)	0.589 (se = 0.031)	0.634 (se = 0.041)
N stage (0/1/2)	0.673 (se = 0.032)	0.673 (se = 0.039)
TNM stage (1/2/3/4)	0.724 (se = 0.029)	0.700 (se = 0.034)
METs (< 4/≥ 4)	0.596 (se = 0.034)	0.600 (se = 0.041)
Age+ T stage+ N stage+ METs	0.730 (se = 0.035)	0.737 (se = 0.038)

C-index, concordance index.

Moreover, we determined the presence of METs through IF staining in 94 CC patients as the validation cohort. Consistent with the results of the training cohort, presence of METs was higher in CC tissues and was a significant prognostic indicator for CC patients in the validation cohort (**Figures 1F–G** and **Supplemental Figure 1D**). The prognostic model including age, T stage, N stage, and METs also showed the highest C-index (0.737) (**Table 2**). The calibration plot presented excellent prognostic values and centralized mainly in the 10% margin of error for 5-year predictive overall survival rate (**Figure 1H**).

### Correlation Between METs With Clinicopathological Features and Inflammatory Markers

**Figure 2:**
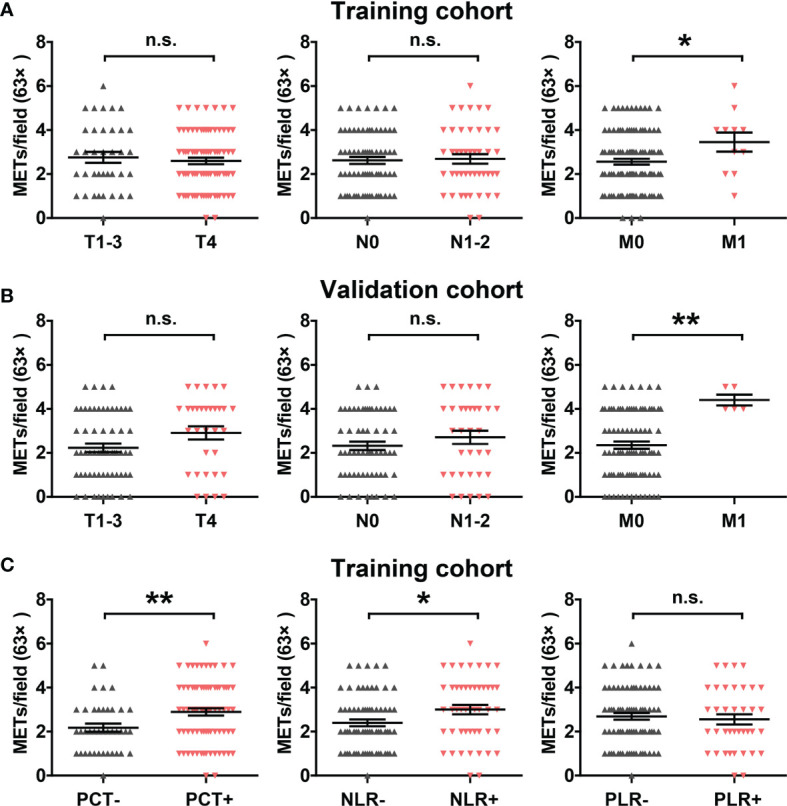
Correlation between METs with clinicopathological features and inflammatory markers. **(A)** Correlation between METs levels with T stage, N stage and M stage in the training cohort. **(B)** Correlation between METs levels with T stage, N stage and M stage in the validation cohort. **(C)** Correlation between METs levels with inflammatory markers PCT, NLR and PLR in the training cohort. * represents p<0.05, ** represents p<0.01. In **(A–C)**, data were calculated by student’s t test. "n.s." means nonsense.

We then analyzed the correlation between METs formation and clinicopathological features in the training cohort and validation cohort. High METs levels were significantly associated with distant metastasis, but not tumor local invasion and lymph node metastasis (**Figures 2A, B**). Meanwhile, considering the close relationship between METs with inflammation, we analyzed the correlation between METs formation and the systemic markers of inflammation, including procalcitonin (PCT), neutrophil-to-lymphocyte ratio (NLR) and platelet-to-lymphocyte ratio (PLR). Patients with abnormally elevated PCT and NLR levels showed the higher levels of METs (**Figure 2C**).

### Crosstalk Between CC Cells and METs *In Vitro*

**Figure 3:**
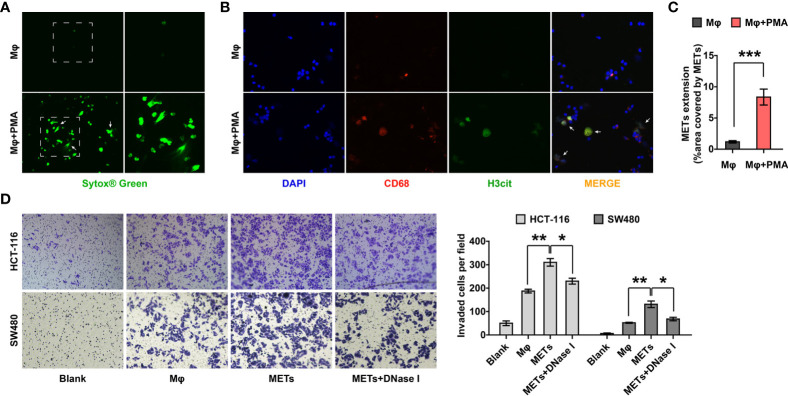
METs promoted the invasion of CC cells. **(A, B)** Representative images of SYTOX^®^ green **(A)** and IF **(B)** staining of METs formation. Arrows indicated the presence of METs. **(C)** Quantification of METs proved that PMA stimulation induced the formation of METs. **(D)** Invasion of HCT-116 and SW480 cells were detected with transwell assay after co-culturing with macrophages or METs, with or without DNase I treatment. * represents p<0.05, ** represents p<0.01, *** represents p<0.001. In **(C, D)**, data were calculated by student’s t test.

Given that the presence of METs in the two cohorts was significantly related to the distant metastasis and poor prognosis of CC patients, we tried to investigate whether METs promoted the metastatic ability of colon cancer *in vitro*. We first detected METs formation induced by PMA stimulation, through SYTOX^®^ green (**Figure 3A**) and IF staining (**Figure 3B**). Results showed that 12 hours of stimulation with 5 μM PMA induced the formation of METs (**Figure 3C**). Then, we used transwell chambers to co-culture CC cells HCT-116 and SW480 (upper chamber) with macrophages or METs (lower chamber). DNase I was introduced to inhibit METs. As expected, METs significantly promoted the invasion of HCT116 and SW480 cells, while DNase I treatment reversed it (**Figure 3D**).

**Figure 4:**
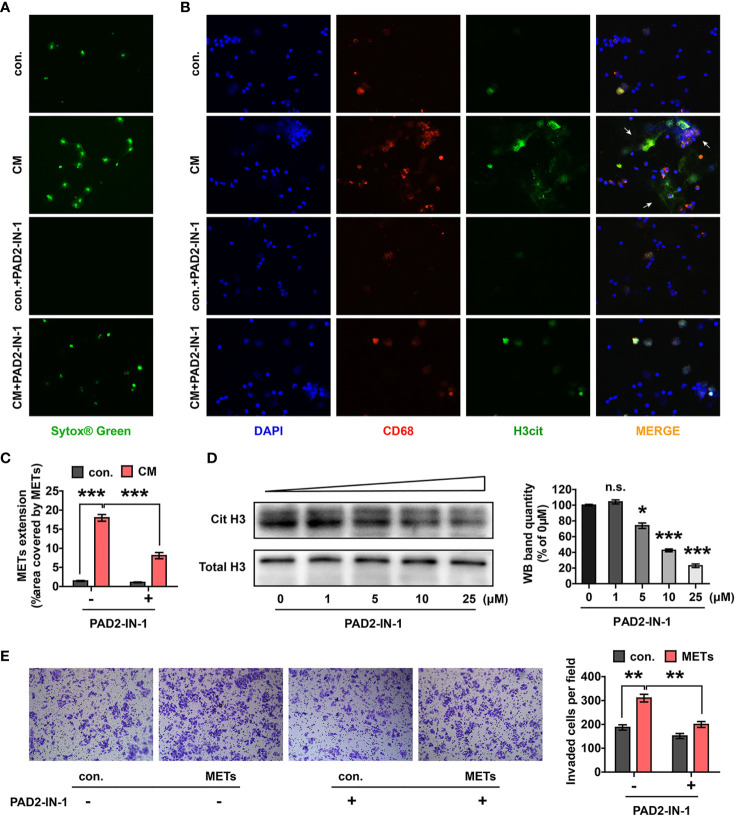
CM of CC cells induced METs formation and the crosstalk between CC cells and METs could be destroyed by PAD2-IN-1. **(A, B)** Representative images of SYTOX^®^ green **(A)** and IF **(B)** staining of METs after the stimulation of HCT-116 cells CM with or without PAD2-IN-1. Arrows indicated the presence of METs. **(C)** Quantification of METs proved that CM stimulation induced the formation of METs, which could be destroyed by PAD2-IN-1. **(D)** Western blotting assay confirmed that PAD2-IN-1 inhibited histone H3 citrullination of macrophages. **(E)** Invasion of HCT-116 cells were detected with transwell assay after co-culturing with METs, with or without PAD2-IN-1. CM stimulation enhanced the invasive ability of HCT-116 cells, which could be destructed by PAD2-IN-1. * represents p<0.05, ** represents p<0.01. *** represents p<0.001. In **(C–E)**, data were calculated by student’s t test. "n.s." means nonsense.

Tumor cells also promote the formation of ETs *in vitro* (18). In order to verify whether CC cells promoted METs formation, the conditioned medium (CM) of HCT-116 cells was collected to stimulate macrophages. Results showed that CM significantly promoted the formation of METs (**Figures 4A–C**). These results demonstrated that there might be an interaction network between CC cells and METs, that is, METs could promote the invasion of CC cells and CC cells could enhance the production of METs, in turn.

### Crosstalk Between CC Cells and METs Could Be Destroyed by PAD2-IN-1

Peptidylarginine deiminases (PADs) have been reported to be widely involved in the formation of ETs, of which PAD2 is the only member confirmed to function in METs formation (29). PAD2-IN-1, a selective PAD2 inhibitor, has been confirmed to inhibit the histone H3 citrullination induced by PAD2 *in vitro* (30). We first stimulated macrophages with different concentrations of PAD2-IN-1 to select the optimal concentration of PAD2-IN-1 to inhibit histone H3 citrullination of macrophages. PAD2-IN-1 at 25 μM was determined as the best condition (**Figure 4D**). After that, HCT-116 cell CM with or without 25 μM PAD2-IN-1 was used to stimulate macrophages and the formation of METs was detected. We found that PAD2-IN-1 significantly inhibited METs formation induced by CM (**Figures 4A–C**). Moreover, we introduced PAD2-IN-1 to the co-culture system of METs and HCT-116 cells to investigate whether PAD2-IN-1 inhibited METs-induced invasion of CC cells. Results showed that the improvement of CC cells invasion induced by METs was significantly reversed by PAD2-IN-1 (**Figure 4E**). The above data supported that PAD2-IN-1 inhibited the crosstalk between CC cells and METs *in vitro*, which should be emphasized in CC therapy.

### Therapeutic Effect of PAD2-IN-1 *In Vivo*

**Figure 5:**
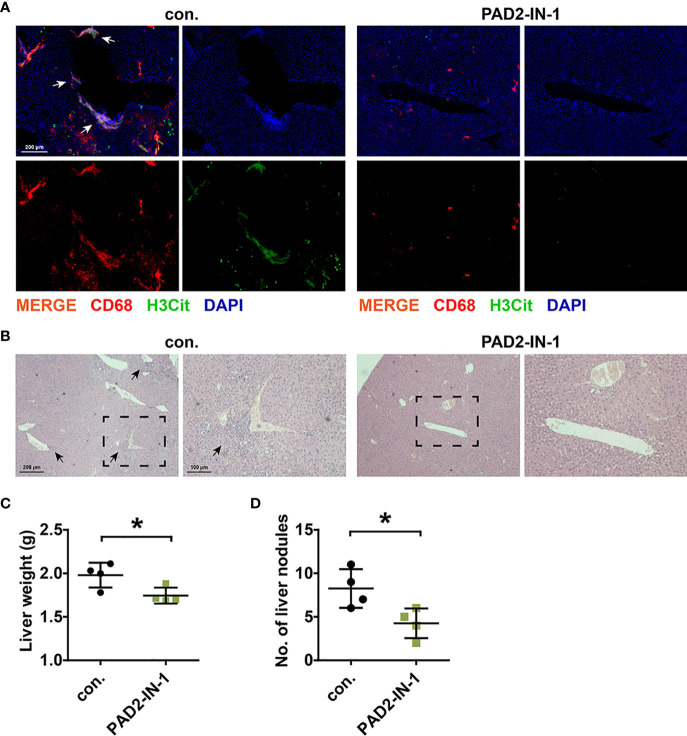
PAD2-IN-1 inhibited METs formation and colon cancer liver metastasis *in vivo*. **(A)** Representative images of IF staining of METs in metastasis lesions in liver. Arrows indicated the presence of METs. **(B)** Representative images of H&E staining of metastasis lesions in liver. Arrows indicated the metastasis lesions. **(C)** Livers were weighted to assess the tumor burden of liver metastatic foci. **(D)** Number of metastasis nodules in livers of mice. * represents p<0.05. In **(C, D)**, data were calculated by student’s t test.

Experiments *in vivo* were further performed to verify the crosstalk between CC cells and METs and the therapeutic value of PAD2-IN-1 *in vivo*. To eliminate the interference of NETs, we first use the anti-mouse Ly6G antibody to deplete neutrophils as described (23). After that, we inoculated MC-38 cells into the tail vein of BALB/c mice and treated the mice with PAD2-IN-1. IF staining was performed to detect the presence of METs and H&E staining was performed to confirm that the nodules were formed by CC cells. We found that CC cells were mainly colonized around blood vessels and were surrounded by METs (**Figures 5A, B**). PAD2-IN-1 not only inhibited METs formation (**Figure 5A**) but also reduced the liver metastasis of CC cells (**Figures 5B–D**), which was consistent with results of *in vitro* experiments. Besides, our results also suggested that PAD2-IN-1 treatment suppressed macrophage infiltration in the mice livers. This finding was consistent with previous studies on the correlation of PAD2 with the infiltration of inflammatory cells and macrophage activation, which revealed the multiple roles of PAD2 in the regulation of tumor immune microenvironment (31, 32).

In conclusion, we found that numbers of METs were high in CC tissues and were correlated with poor prognosis and distant metastasis of CC patients. Through *in vitro* and *in vivo* experiments, we confirmed that there was a positive feedback loop between CC cells and METs, which could be blocked by PAD2-IN-1 inhibitors. This discovery filled the research gap of METs in the field of CC, identified a new mechanism of macrophages in the progression of CC, and provided new ideas and potential therapeutic targets for the immunotherapy of CC patients, especially patients with distant metastasis.

## Discussion

The development of malignancies relies on a complex tissue environment referred to as the tumor microenvironment. This environment is critical for tumor growth, metastasis, and tumor-associated angiogenesis (33). Numerous tumor cells, mesenchymal cells, and immune cells as well as lymphatic vessels and blood vessels constitute the tumor microenvironment, which may be conducive to the occurrence and development of tumors (34). Studies have shown that inflammation has a close relationship with tumors. The tumor microenvironment composed of main inflammatory cells is a necessary participant and supporter of several tumor biological behaviors (35). Among these inflammatory cells, tumor-associated macrophages (TAMs) and tumor-associated neutrophils (TANs) account for a large proportion. TAMs are one of the major immune cells that infiltrate the tumor and are the main producers of multiple inflammation mediators (such as chemokines), which contribute to the activation and maintenance of chronic inflammatory processes (10). At present, increasing numbers of studies have confirmed that tumor-infiltrating macrophages and domestication of tumor microenvironment by macrophages are essential in tumor development. In the tumor microenvironment, macrophages can express pro- or anti-tumoral functions. This plasticity is a characteristic of the monocyte-macrophage system and reflects the particularity of these cells (11).

CC is a malignant tumor with abundant macrophage infiltration (36, 37). However, the role of macrophages in the progression of CC is still controversial. Some studies showed that high macrophage infiltration along the tumor front brings a high survival advantage for patients with CRC (38). However, more studies demonstrate that TAM promotes the growth and development of tumors (39, 40). These findings suggest that there may be other factors that function in the crosstalk of macrophages and colon cancer cells.

METs formation is a special death form of macrophages. The main components of METs, roughly the same as NETs, include extracellular fibers composed of DNA extending outside the cell boundary, which can be degraded by DNase I or micrococcal nuclease treatment. By staining the known ET components (such as citrullinated histone and elastase), the formation of METs is determined (13). METs can be induced by PMA, LPS, TNF-α, and other factors, and participate in the response to bacteria and toxins, as well as pathological processes such as acute kidney injury (20–23). In tumor research, METs have only been reported as closely related to the poor prognosis of patients with nonfunctional pancreatic neuroendocrine tumors. However, the clinical significance and molecular mechanisms of METs in CC are still lacking. Our study confirmed for the first time that presence of METs was involved in the progression of CC and affected the survival outcome of CC patients. Through immunofluorescence assays, we found that macrophage infiltration was not significantly related to the prognosis of patients, while METs formation was an independent prognostic of CC patients. High METs predicted the worst prognosis for CC patients. This result was consistent with a previous study showing that macrophages participate in the immune response of nonfunctional pancreatic neuroendocrine tumors in the form of METs (24). However, they did not confirm this breakthrough conclusion through *in vivo* and *in vitro* experiments.

The potential value of METs in the clinical diagnosis and treatment of CC patients may be huge. Given that we have confirmed the critical role of METs in CC, we conducted further *in vitro* experiments using CC cell lines. Through transwell assays, we found that the co-culture of PMA-induced METs with CC cells promoted the invasion of CC cells. Additionally, the CM of CC cells also enhanced the formation of METs. This finding confirmed that there was a positive feedback between METs and CC cells. They promoted each other and together led to tumor progression and poor prognosis of CC patients. Cytokines including Interferon γ (IFN-γ), tumor necrosis factor α (TNF-α), and interleukin 8 (IL-8) have been confirmed to induce METs formation (13, 41). IL-8 and TNF-α can be secreted by CC cells and correlate with CC progression, which may be the molecular basis for the formation of METs induced by CC cells (42, 43). Further studies are needed to elucidate the cytokines and the regulation mechanisms involved in the process of CC cells-mediated METs formation.

Peptidylarginine deiminases (PADs) catalyze the conversion of positively charged arginine residues to neutrally charged citrulline. The N-terminal tails of H3 and H4 histones are the main targets of PADs because of their arginine-richment (44). PADs family consists of 5 members: PAD1, PAD2, PAD3, PAD4 and PAD6. PAD2 and PAD4 have been clearly confirmed to regulate NETs release (44–46), but the role of PAD2 and PAD4 in METs is controversial. A study finds that PAD2 mRNA is strongly expressed while PAD4 expression is very low in RAW 264.7 mouse macrophages. PAD2 expresion is significantly related with METs formation (29). However, another study confirms the positive expression of PAD4 in mouse macrophages from lymphoid tissue, which promoted the formation of METs (47). Besides, some studies show that not all observed METs are composed of histones and are generated in a PAD-dependent manner. METs can also be released independent of PAD enzymes-mediated histone citrullination (41, 48). In this study, we mainly focused on PAD2 and confirmed for the first time that PAD2-IN-1, an inhibitor of PAD2, inhibited the interaction between METs and CC cells. More and more tumor-targeted drugs are not designed to destroy tumor cells, but to block the interaction between tumor cells and other cells, such as PD-1/PD-L1 monoclonal antibodies and anti-VEGF bevacizumab. Our research focused on the positive feedback between METs and CC cells. This discovery fills the research gap of METs in the field of CC, described a new mechanism of macrophages in the progression of CC, and provided new ideas and potential therapeutic targets for the immunotherapy of CC patients, especially patients with distant metastasis.

## Data Availability Statement

The original contributions presented in the study are included in the article/**Supplementary Material**. Further inquiries can be directed to the corresponding author.

## Ethics Statement

The studies involving human participants were reviewed and approved by the Ethics Committee of Qilu Hospital of Shandong University. The patients/participants provided their written informed consent to participate in this study. The animal study was reviewed and approved by the Ethics Committee of Qilu Hospital of Shandong University.

## Author Contributions

CL, XQ and TC contributed to conception and design of the study. TC and YW carried out our research. ZN and TZ collected the specimens and perform the follow-up. CL and JW participated in data analysis and interpretation. TC and CL wrote the manuscript. All authors contributed to manuscript revision, read, and approved the submitted version.

## Funding

Our study was supported by National Natural Science Foundation of China (Grant No. 81372810).

## Conflict of Interest

The authors declare that the research was conducted in the absence of any commercial or financial relationships that could be construed as a potential conflict of interest.

## Publisher’s Note

All claims expressed in this article are solely those of the authors and do not necessarily represent those of their affiliated organizations, or those of the publisher, the editors and the reviewers. Any product that may be evaluated in this article, or claim that may be made by its manufacturer, is not guaranteed or endorsed by the publisher.

## Abbreviations

CRC, colorectal cancer; CC, colon cancer; RC, rectal cancer; mCRC, metastatic colorectal cancer; OS, overall survival; TAMs, tumor-associated macrophages; TANs, tumor-associated neutrophils; ETs, extracellular traps; METs, macrophage extracellular traps; NETs, neutrophil extracellular traps; PAD2, peptidylarginine deiminase 2; IF, immunofluorescence staining; H3Cit, Citrullinated histone H3; PMA, phorbol 12-myristate 13-acetate; CM, conditioned medium; C-index, concordance index; PCT, procalcitonin; NLR, neutrophil-to-lymphocyte ratio; PLR, platelet-to-lymphocyte ratio.
