# Supplementary material for: Interaction Between Macrophage Extracellular Traps and Colon Cancer Cells Promotes Colon Cancer Invasion and Correlates With Unfavorable Prognosis
Source: Front Immunol. 2021 Dec 1;12:779325. doi: 10.3389/fimmu.2021.779325 (PMC8671452; doi:10.3389/fimmu.2021.779325)
Supplement: Supplementary file 1 [file DataSheet_1.pdf]

Supplementary Material

Supplementary Figure 1.

(A) In the training cohort, macrophage infiltration (left panel) and METs (right panel) were higher expressed in CC tissues than in para-tumor tissues.

(B) The correlation between the number of METs and macrophages infiltration was analyzed using student's t test.

(C) The correlation between the number of METs and macrophages infiltration was analyzed using Chi-square test.

(D) In the validation cohort, macrophage infiltration (left panel) and METs (right panel) were higher expressed in CC tissues than in para-tumor tissues.

*** represents p<0.001. N.s. meas nonsense. In (A), (B) and (D), data were calculated by student's t test.

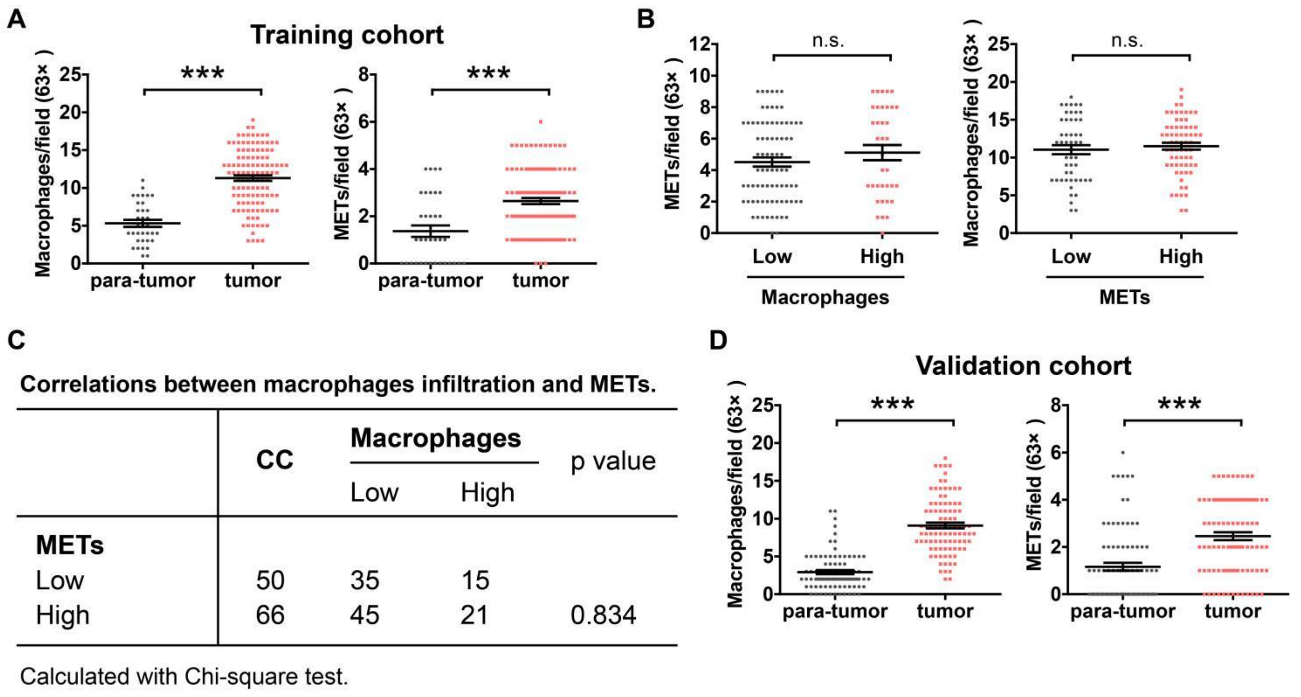

Supplementary Figure 1

Supplementary Table 1. Clinicopathological characteristics of patients.

Patient characteristics	Training cohort	Validation cohort
Age (median, < 60/≥ 60), years old	64 (41/75)	65 (28/66)
Gender (male/female)	77/39	48/46
Tumor size (< 5/≥ 5), cm	55/61	41/53
T stage (1/2/3/4)	6/8/23/79	1/10/51/32
N stage (0/1/2)	71/27/18	60/25/9
M stage (0/1)	105/11	89/5
TNM stage (1/2/3/4)	12/54/39/11	1/48/31/5
Death (no/yes)	61/55	55/39
